# Validation of the Spanish version of the Franciscan Hospital for Children Oral Health-Related Quality of Life questionnaire

**DOI:** 10.4317/medoral.22553

**Published:** 2018-09-28

**Authors:** Virginia Rollon-Ugalde, Jose-Antonio Coello-Suanzes, Antonio Castaño-Seiquer, Emilio Lledo-Villar, Ivanna Espinoza-Visval, Ana-Maria Lopez-Jimenez, Pedro Infante-Cossio, Angel Rollon-Mayordomo

**Affiliations:** 1 Department of Oral and Maxillofacial Surgery, Virgen Macarena University Hospital, Seville; 2 Department of Preventive and Community Dentistry, Faculty of Dentistry, University of Seville, Seville; 3 Department of Experimental Psychology, Faculty of Psychology, University of Seville, Seville; 4 Department of Surgery, School of Medicine, University of Seville, Seville, Spain

## Abstract

**Background:**

The Franciscan Hospital for Children Oral Health-Related Quality of Life questionnaire (FHC-OHRQOL-Q) is an instrument designed specifically for parents and caregivers of patients with special needs that has not yet been applied in Spain. The aim of this study was to adapt it to Spanish and evaluate its reliability and validity in patients with intellectual disability (ID) treated under general anesthesia.

**Material and Methods:**

The study was conducted in two different stages: a) cross-cultural adaptation of the original questionnaire, and b) cross-sectional study on 100 parents and caregivers who completed the piloted FHC-OHRQOL-Q. The patients were examined according to the WHO methodology. Dental treatments performed were recorded. Statistical tests were used to evaluate reliability (internal consistency) and validity (content, criterion, construct and discriminant) of the instrument.

**Results:**

The mean age was 24 years (range=4-71 years). The most frequent causes of ID were psychomotor retardation (25%) and cerebral palsy (24%). The items most frequently answered by parents and caregivers were eating and nutrition problems (80%) and bad breath/taste (57%). Reliability (Cronbach’s alpha coefficient) was considered excellent (alpha=0.80-0.95). The analysis of the factorial validity yielded similar results to the original questionnaire. The high response rate of items (>96%) allowed content validity. Criterion validity was confirmed by a significant correlation with questions on oral health and oral well-being. Discriminant validity was demonstrated by the significant association of ≥21.5 years of age with worse oral symptoms (*p*=0.034) and parental concerns (*p*=0.005), DMFT index ≥3 with daily life problems (*p*=0.02), ≥4 decayed teeth with daily life problems (*p*=0.001), and >2 dental extractions with oral symptoms (*p*=0.000), daily life problems (*p*=0.002) and parent´s perceptions (*p*=0.043).

**Conclusions:**

The FHC-OHRQOL-Q in Spanish is a reliable and valid instrument to apply in clinical practice to evaluate the impact of OHRQOL in mostly adult patients with ID, accessible to Spanish-speaking parents and caregivers.

** Key words:**Oral health-related quality of life, intellectual disability, cross-cultural validation, psychometric properties, validation, questionnaire.

## Introduction

Patients with intellectual disability (ID) are generally considered to have special health care needs (SHCN). Maintaining good oral health in these patients can be a challenge due to problems and limitations for their collaboration in the dental office, both for clinical examination and for dental treatment (1,2). As a result, these patients usually have worse oral health due to lack of dental care, faster progression of oral disease and restricted access to adequate dental treatment (2,3). In addition, in many of them, dental treatment can only be performed under general anesthesia (GA), which increases consumption of resources, cost of dental care (1) and risk associated with the anesthesia procedure (4,5).

The concept of quality of life related to oral health (OHRQOL) refers to the oral health or disease of an individual in relation to parameters such as daily function, well-being and social interaction (6). It is a subjective self-evaluation of the patient that requires specifically designed and validated questionnaires with different methodologies whose results attempt to reflect the effectiveness of the dental treatment (2). However, self-assessment of quality of life (QOL) in patients with SHCN is difficult due to their deficiency in communication skills, so usually the measurement of their OHRQOL will require an instrument that must be handled by their parents or caregivers.

The evaluation of the impact of dental treatment performed under GA using OHRQOL questionnaires (OHRQOL-Q) has mainly focused on healthy children and cerebral palsy (CP) and autism patients (7-15). Studies on OHRQOL in adults with ID are very scarce and have been carried out with not validated qualitative questionnaires (5) or validated questionnaires for children (2). The Franciscan Hospital for Children Oral Health-Related Quality of Life questionnaire (FHC-OHRQOL-Q) is a tool designed specifically for parents and caregivers of children with ID created by Baens-Ferrer *et al.* (16) in Boston (USA). It is an increasingly used instrument that has shown that dental treatment under GA in a child population improves the QOL of children with SHCN and their families (14,16,17). However, the validation characteristics of the original questionnaire were not provided and, to our knowledge, neither has it been applied in the Spanish population nor has it been adapted to the Spanish language, even though the creators used a preliminary version in our language.

Taking this scenario into account, the aim of this study was to transculturally adapt the original FHC-OHRQOL-Q devised by the authors in 2005 (16) to our linguistic and cultural milieu. The objective was to assess the psychometric characteristics of the piloted instrument in a group of patients with ID treated under GA in our clinical setting, and to evaluate its validity and reliability to provide not only a specific and useful tool to estimate the impact on their QOL, but also to detect their dental treatment needs.

## Material and Methods

-Study stages. The present study was conducted on two different stages: 1) cross-cultural adaptation of the FHC-OHRQOL-Q in a pilot group and, 2) cross-sectional study in a control group to evaluate its reliability and validity. It comprised patients with ID referred to the Department of Oral and Maxillofacial Surgery, Virgen Macarena University Hospital, Seville (Spain), to receive dental treatment under GA who came accompanied by their parents or responsible caregivers. Before starting any step in the process of adaptation, the main researcher contacted the creators of the questionnaire by email, obtained their consent for the development of the present study and invited them to participate in it. The study was approved by the Ethical Committee of the Hospital.

At the first visit, parents and caregivers were informed of their participation in the study, received the questionnaire and written information about the study characteristics. Those parents and caregivers who were fluent in Spanish and willing to participate in the study signed informed consent.

For cross-cultural adaptation, two researchers and two caregivers reviewed the original version in English and the preliminary version in Spanish to analyze the differences and contradictions between both versions. The items were reviewed and modified to resolve discrepancies. After adding some words to improve understanding, equivalence and sociocultural adaptation, the resulting new Spanish version only presented minor changes. Next, a comprehension and equivalence analysis of this final questionnaire was carried out in a pilot group of 20 parents and caregivers. In the second stage, the field survey was conducted on a control sample of 100 consecutive patients admitted to our Department between 2012 and 2016.

- Procedures. Prior the initiation of dental treatment, the parents or caregivers completed the FHC-OHRQOL-Q in the presence of an interviewer who only helped them if they had difficulty in understanding the questions but did not indicate or explain the answers.

Under GA, two researchers performed a full oral examination using an operating light, a three-in-one syringe to dry the teeth, flat-mouth mirrors and periodontal probes. Dental status, caries, periodontal status, tooth loss and DMFT (decay-missing-filled teeth) index/dft (decay-filled teeth) index were recorded according to the WHO methodology (18). Fillings, extractions, scaling and root planing and endodontic treatments were also recorded.

-Questionnaire. The FHC-OHRQOL-Q is a questionnaire that gathers information related to the impact that oral problems can have on the QOL and the general well-being in individuals with SHCN. It is a questionnaire to be completed by parents or caregivers, which comprises 41 items distributed in four sections/dimensions (D) (16): D1 (oral symptoms), D2 (daily life problems) and D3 (parenteral concerns), having each question six possible answers with a numeric value of 0-5. The answer and its value are: never value 0, hardly ever value 1, once in a while value 2, some of the time value 3, most of the time value 4, and all of the time value 5. D4 consists of four questions to assess the parent´s perception of the QOL by a Visual Analog Scale 0-10 (VAS). The score of each dimension is obtained from the mean of its items. The higher the score obtained in the questionnaire, the worse OHRQOL of the patient will be.

-Analysis. In the first stage of cross-cultural adaptation to Spanish, comprehension was estimated through the detection of non-answered questions and the free text comments that parents and caregivers wrote at the end of the questionnaire. Reliability test-retest was obtained by Pearson correlation. In the second stage of cross-sectional study, the following psychometric characteristics were analyzed:

The content validity was evaluated based on the consensus of the researchers participating in the study, the assurance that the questionnaire was originally designed following other similar questionnaires, the response rate, and the verification that all responses were answered (sensitivity of scale).

The criterion validity was evaluated by comparing the results of each dimension with a descriptor related to the OHRQOL used as proxy (2), since there is no universal gold standard for QOL measurements (19,20). We used two of the questions from D4 of the questionnaire itself: Q1 (“What is your opinion of the appearance of your child’s teeth and mouth?”) and Q3 (“How do you feel about your child’s overall oral well-being?”).

The discriminant validity was measured by relating each dimension with the age of the patients, dental status and procedures performed. These variables are associated with oral health and disease progression. We used the Pearson correlation coefficient (r) and the comparison of groups of patients formed from the median of each clinical variable (8). If the median did not detect any statistical difference, we used the 25th or 75th percentile.

The construct validity was analyzed through an exploratory factorial analysis of the main components of the instrument and oblimin direct rotation in which the 37 items of the first three dimensions were included and excluded the four of the last dimension. The objective was to identify the emerging and underlying factors that become evident when we attempted to group the items answered by parents and caregivers. Reliability was defined as the internal consistency of the dimensions, calculated according to the standardized Cronbach’s alpha coefficient. The expected alpha coefficient was estimated at 0.70.

For each item and dimension, we analyzed the frequency of responses. The values were presented as mean (M), median (Md), range, interquartile range and standard deviation (SD). For the non-answered questions, the mean of the other items was imputed. The prevalence of symptoms was defined as the percentage of patients who responded “some of the time”, “most of the time” or “all of the time” (16).

The sample size was calculated to detect a correlation between the dimensions and the clinical variables, on the assumption that it would be weak (r≤0.3). With an alpha error of 0.05 and a beta error of 0.2, the sample required 84 subjects. Student t test, Mann-Whitney U test, ANOVA and Pearson´s correlation coefficient were used to analyze the items and to obtain evidence of validity and reliability. A value of *p*<0.05 was considered statistically significant. The data was analyzed using the statistical package SPSS V.22.

## Results

The pilot study of cross-cultural adaptation revealed that none of the parents and caregivers reported greater difficulty in understanding the questionnaire, although five of them left non-answered items due to lack of knowledge. The test-re-test reliability was confirmed using the Pearson correlation, showing a value of 0.96, 0.98, 0.75 and 0.95, respectively for each dimension.

The cross-sectional study was conducted on 100 patients who met the inclusion criteria and whose parents and caregivers answered the questionnaire between June 2012 and June 2016. The mean age of the patients was 24 years (range=4-71, M=24.2, SD=13.1). 59 of them were males and 41 females. The causes of ID were psychomotor retardation (25), cerebral palsy (24), developmental disorder (23), rare diseases (18), Down syndrome (7) and psychiatric disorder (3). 76 of caregivers were family-members (66 mothers, 4 fathers, 4 both, 1 grandmother and 1 brother), while 24 patients lived in a residence, so their caregivers were the workers in their residence responsible for them. The final version of the questionnaire was completed by 74 caregivers, 14 did not answer less than 10 items, and 12 more than 10 items. All the items had a response rate higher than 96%. Non-answered or lost items accounted for 7% of the questionnaire, with the “pain” of D1 being the most frequent.

The oral examination showed a DMFT index of 3.4 (SD=3.5). 82% of the patients presented decayed teeth (M=4.2, SD=3.5). In total 555 dental procedures were performed (M=5.5, SD=3.6) consisting of 245 fillings (M=2.45, SD=2.52) and 239 dental extractions (M=2.39, SD=3.06). Scaling and root planing and endodontic treatments were performed in 54 and 17 patients respectively.

The measurement of the content validity using the scale of sensitivity revealed that all the possible items were answered except for four items of D1 and five of D2 that did not reach the maximum value. In  Table 1, the values of central tendency and dispersion of the four dimensions of the questionnaire are shown. The most frequent items that parents and caregivers responded were eating and nutrition problems (80%), bad breath/taste (57%), anger about oral problems (56%), miss of sleep (46%), bleeding with brushing (41%), disrupt family life (39%) and toothache (38%).

**Table 1:**
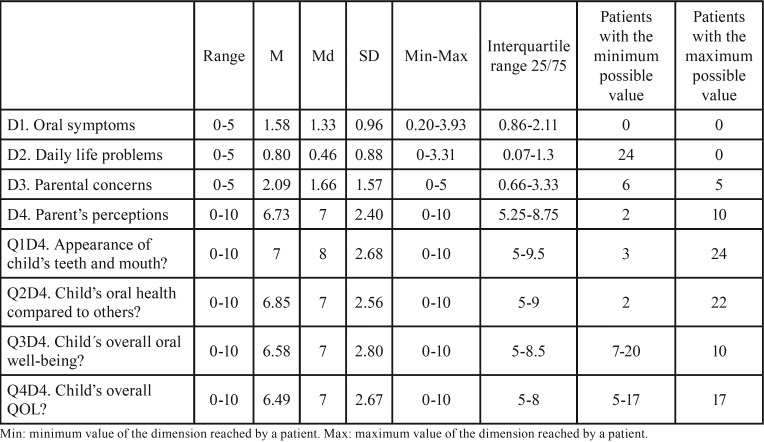
Measurement of central tendency and dispersion of the four dimensions and extreme values distribution.

The criterion validity was confirmed by a significant correlation of Q1D4 and Q3D4 with the first three dimensions, obtaining the highest coefficients between Q3D4 and D1 (r=0.39) and D2 (r=0.45).

The results of discriminant validity are shown in  Table 2. D1 showed a significant correlation with age (*p*=0.038), DMFT index (*p*=0.03), number of treatments (*p*=0.03) and number of dental extractions (*p*=0.007), D2 with DMFT index (*p*=0.04), number of decayed teeth (*p*=0.03), number of treatments (*p*=0.04) and number of dental extractions (*p*=0.022), and D4 with number of extractions (*p*=0.033). The analysis of the dimensions according to the selected cut-off points revealed that patients ≥21.5 years of age showed worse D1 (*p*=0.034) and D3 (*p*=0.005), patients with DMFT index ≥3 worse D2 (*p*=0.028), patients with ≥ 4 decayed teeth worse D2 (p=0.01), and patients undergoing >2 dental extractions worse D1 (*p*=0.000), D2 (*p*=0.002) and D4 (*p*=0.043) ( Table 3).

**Table 2:**
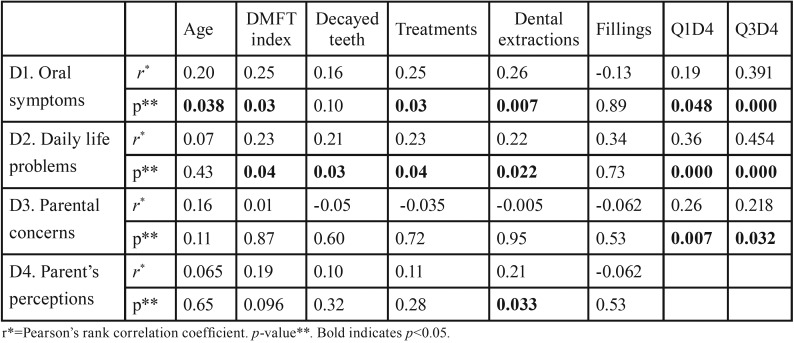
Pearson correlations and p values of dimensions scores with OHRQOL and clinical variables.

**Table 3:**
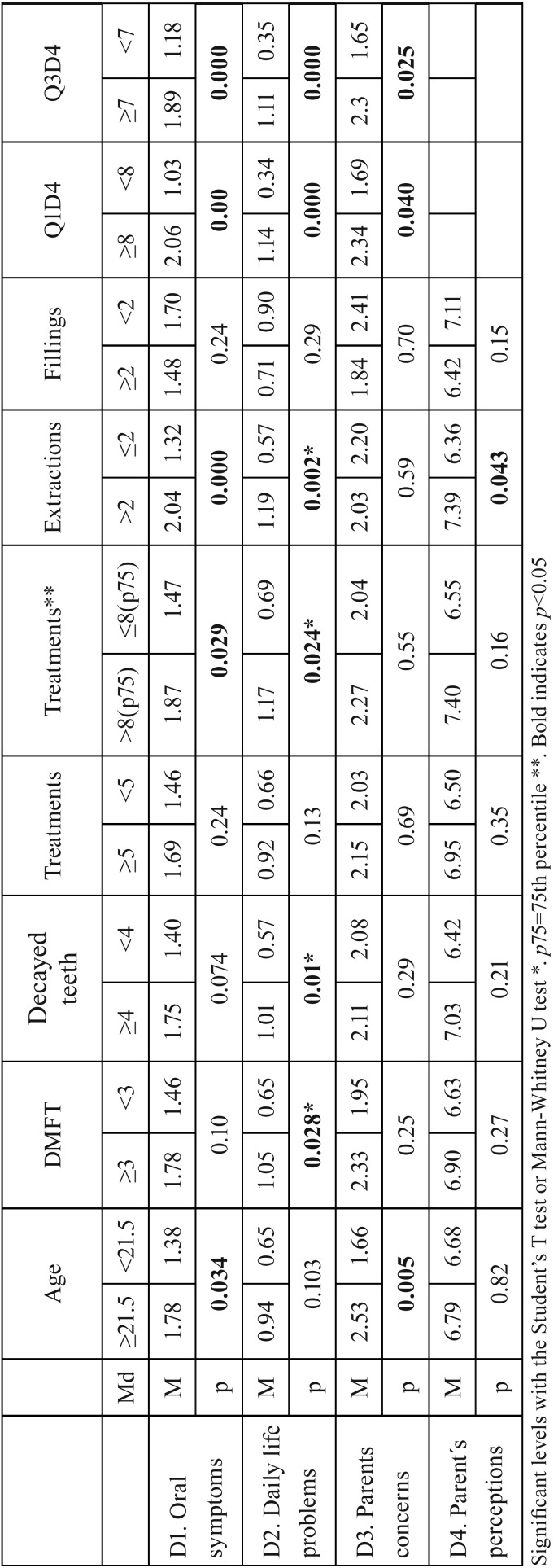
Values of the dimensions according to the cut-off points of medians or percentiles for each clinical variable.

Reliability measured by the Cronbach alpha coefficient showed adequate consistency that ranged between 0.80 and 0.95. We calculated the Cronbach alpha coefficient excluding in each case one of the items and obtained lower reliability levels for each item. Cronbach’s alpha was very similar in the factorial analysis.

We performed a factorial analysis with Oblimin direct rotation and the Kaiser-Meyer-Olkin measure of sampling adequacy (KMO-test). The results of the KMO-test index (0.84) and Bartlett sphericity proof (χ2 = 3025.50, *p*<0.001) revealed the suitability of the matrix correlation for factorial analysis. The factorial analysis with Varimax rotation and factorial extraction with an eigenvalue >1 obtained by the retention criterion of the Kaiser factor corresponded to seven factors that explained 86.17% of the factorial variance. The first three factors explained 71.92% of the variance while the remaining four 8.62%. Given the accumulation of variance described by the first three factors, we conducted a second factorial analysis to provide greater consistency and simplicity for the use of the instrument in clinical settings. The same procedure of factorial extraction and rotation was followed, maintaining only three factors and using for its interpretation the items with factorial values greater than 0.5.  Table 4 shows the configuration matrix with the three proposed factors.

**Table 4:**
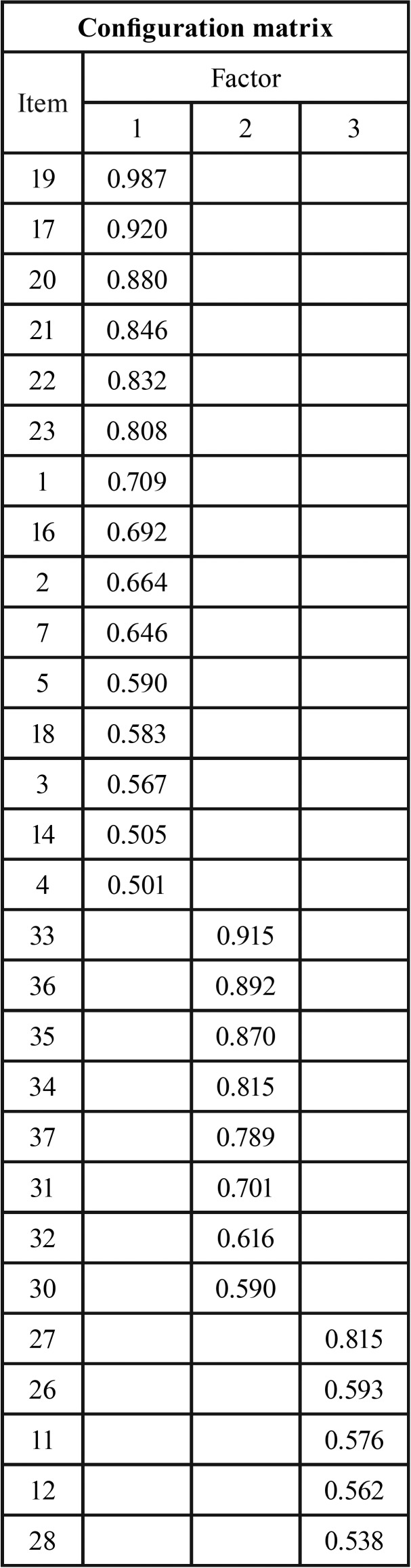
Analysis of the main components of the FHC-OHRQOL-Q using rotated component matrix (factor extraction method with analysis of main components and Kaiser Oblimin rotation).

## Discussion

The results of our study suggest that the Spanish version of the FHC-OHRQOL-Q is valid and equivalent to the original English questionnaire and presents similar reliability and validity, as well as analogous limitations.

The Cronbach’s alpha coefficients of the Spanish version of the FHC-OHRQOL-Q were similar to those of the original questionnaire with values ranging from 0.80 to 0.95. As reliability exceeds 0.7, this level was considered adequate to make comparisons and check the OHRQOL in each individual. It showed a degree of internal consistency similar to that obtained by means of other questionnaires which comprise the same number of items (18). All this confirms that the internal validity of the questionnaire has not been altered by the translation process.

We used a factorial analysis in order to validate the instrument in terms of size, sampling technique, language and culture. The sampling technique was non-probabilistic, since it is not a requirement for validation. Our sample was very different from the original study by Baens-Ferrer *et al.* (16), although both samples were heterogeneous and included different types of patients. However, since the results obtained were similar to those of the original questionnaire, we can affirm that both questionnaires measured the same, despite the differences between the samples. The mean of the first three dimensions was slightly worse in our study compared to the original study (16), which can be explained by the higher mean age and worse oral health of the patients in our series, since 100% corresponded to patients with ID mostly adults. The results observed in the analysis of the factorial groups also confirmed the hypothesis that cross-cultural adaptation did not alter the questionnaire. The analysis of the Cronbach alpha coefficient demonstrated its reliability also for each isolated item.

Exploratory factor analysis detected three dimensions (or specific factors) that integrate the QOL with elements such as pain, image and environmental consequences. These three factors were compatible with the first three dimensions of the FHC-OHRQOL-Q. The first factor referred to the symptoms related to oral health, the second to parenteral reports, and the third to daily life symptoms and the socio-functional impact of oral health.

The apparent validity, referred to the degree to which this instrument served to measure what was designed, was based on the fact that the original questionnaire was created following other similar questionnaires (16). Oral symptoms were comparable to those presented in the COHIP (2), COQOL (21) and ECOHIS (9) questionnaires. The problems of daily life consisted of questions about daily, social functions, and emotional well-being, such as the COHIP (2) and ECOHIS (9) questionnaires, and the Malden *et al.* (8) questionnaire. The parenteral concerns and the parent’s perception dimensions were equivalent to the FIS questionnaire (2,8) and to the dimensions of “parental distress” and “family function” of the ECOHIS questionnaire (9).

The content validity was confirmed by consensus of the researchers and the high individual response rate. Although none of the parents and caregivers mentioned great difficulty in understanding the questionnaire, in the pilot study 5 caregivers commented that they did not know the specific symptoms of the patients due to impossible communication with them and 3 doubted whether some questions were addressed to the patient or the caregiver. Therefore, in the final version we added a brief explanatory introduction to those questions. The most frequent non-answered questions of D1 were referred to “pain”, while in D2 were about “smiling”. These questions could be related to problems or feelings of the patients, more difficult to detect by parents and caregivers. The non-answered questions were assessed with the mean of each dimension (2,15,22), avoiding any loss in the population size or an incorrect value bias.

The answers were distributed across the scale in 23 questions. Patients with a maximum or minimum value accounted for 24%, which showed good sensitivity and population variability, unlike other questionnaires (8,9,12). The significant correlation of each dimension with questions Q1D4 and Q3D4 used as proxies ensured the criterion validity as other authors have reported previously (2,23). The significant correlation of clinical variables with some dimensions of the instrument demonstrated its discriminative validity and sensitivity analysis. The coefficient varied between 0.2 (decayed teeth) and 0.26 (dental extractions). Even when the values were not high, they indicated an important correlation since they are variables of a different nature and concordant with other studies (9,21).

In our study, parents and caregivers of patients ≥21.5 years of age reported significantly worse oral symptoms and parenteral concerns than patients <21.5 (M=1.78 vs. M=1.38), probably because oral health worsened as a result of the long evolution of their ID and the absence of previous treatments. These data coincided with a study using the COHIP-14sp questionnaire, which indicated that patients older than 7 years of age had a worse DMFT index and that those older than 30 years of age had a significantly worse OHRQOL (2). Patients with a DMFT index ≥ 3 had significantly more problems of daily life than DMFT index <3 (M=1.05 vs. M=0.65). Although some studies linked the DMFT index with the number of decayed teeth (24), we analyzed this variable independently and found that patients with ≥4 decayed teeth had significantly more daily life problems than <4 decayed teeth (M=1.01 vs. M=0.57), in line with previous studies (6,9,11-13). The number of procedures is indirectly related to a worse OHRQOL and was significantly related to oral symptoms and daily life problems. The dental extractions showed a greater significant correlation (r=0.26) with oral symptoms, and to a lesser extent, with daily life problems and parent´s perception. Patients undergoing >2 dental extractions showed significantly worse oral symptoms than < 2 dental extractions (M=2.04 vs. M=1.32.), oral daily life problems (M=1.19 vs. M=0.57) and parent´s perception (M=7.39 vs. M=6.36). Other studies did not detect significant differences in OHQOL when they analyzed dental extractions or fillings in a categorical way (2,8).

The differences of our results in comparison with previous studies reported in the literature (1,4,8,12,14,22) can be explained by the characteristics of our patients (65% over 14 years of age, all of them with ID), their oral health and the treatments performed, together with the specific psychometric properties of the questionnaire. The communication problems and the reasoning deficit of patients and the limited knowledge of their symptoms by the parents and caregivers are the main difficulties to evaluate the OHRQOL in patients with SHCN (12,25,26). The OHRQOL studies in patients with communication problems have focused on healthy children and CP (8,9,12,21,27). There are only 3 papers on QOL and oral health in the adult population with ID (2,5,21). Of these studies, only one (2) analyses the OHRQOL with a specific questionnaire created for healthy children (28), and there are no questionnaires for adults with ID.

The FHC-OHRQOL-Q translated and adapted to Spanish seems to operate as a specific questionnaire that provides valuable information to evaluate OHRQOL in adult patients with ID in our setting. It can be used first as a diagnostic tool to detect dental treatment needs and provide the appropriate treatment to each patient, and in a second step, as a follow-up instrument after treatment. The cross-cultural adaptation only added a few words to the original questionnaire, and its compressibility and reliability were confirmed by the pilot study. The questionnaire was simple and easy to administer to parents and caregivers, being widely accepted by them, since the translation is understandable and adapted to the Spanish language. Researchers and practitioners have never complained of incomprehensible expressions or difficulties in its use. The limitations of our study are the lack of control of variables related to OHRQOL, such as the type of cognitive deficit, bruxism, convulsions, degree of cooperation, medication, type of diet and caregiver (2,5,12,29,30).

## Conclusion

The results of the present study showed that the FHC-OHRQOL-Q adapted to the Spanish language is a reliable and valid instrument both for research and for application in daily clinical practice. It is a useful questionnaire to detect treatment needs and to evaluate the impact of OHRQOL in mostly adult patients with ID, accessible to Spanish-speaking parents, caregivers and practitioners involved in the treatment and follow-up of these patients.
